# Antibacterial Activity of *Bifidobacterium breve* Against *Clostridioides difficile*

**DOI:** 10.3389/fcimb.2019.00288

**Published:** 2019-08-07

**Authors:** Jingpeng Yang, Hong Yang

**Affiliations:** State Key Laboratory of Microbial Metabolism, School of Life Science and Biotechnology, Shanghai Jiao Tong University, Shanghai, China

**Keywords:** *Bifidobacterium breve*, *Clostridioides difficile*, toxin production, gene expression, cytoplasmic membrane

## Abstract

*Bifidobacterium breve* (YH68) is widely used in the fields of food fermentation and biomedicine. In this study, we explored the antibacterial activity of the cell free culture supernatant (CFCS) of YH68 against *Clostridioides difficile* ATCC 9689 (CD) by measuring multiple indexes, including the growth, spores production, toxin A/B production, and the expression levels of the *tcdA* and *tcdB* genes of CD. In addition, we examined the changes in major cellular functional groups, structures, permeability, integrity, and the proton motive force (PMF) of the cytoplasmic membrane. The results showed that double-dilution ratio of YH68-CFCS (3 × 10^9^ CFU/mL) was the MIC value. The cell density, spores production, and the toxin production of CD treated with YH68-CFCS were lower than that of the control (*p* < 0.05). In addition, the gene expression levels of *tcdA* and *tcdB* in CD treated with YH68-CFCS were significant downregulated (*p* < 0.05). Marked differences were observed in the cell membrane and cell wall by a FT-IR spectroscopy and SEM. Analysis of the cell membrane permeability and integrity of the CD cells revealed that YH68-CFCS induced the leakage of a large amount of intracellular K^+^, inorganic phosphate, ATP, nucleic acids and proteinaceous substances. Furthermore, PMF analysis indicated that there was a significant change in Δψ and ΔpH. These findings demonstrated that the antibacterial activity of YH68-CFCS against CD involved the inhibition of growth, spore production, toxin production, and virulence genes expression; a consumption of PMF in the cytoplasmic membrane, the formation of pore in the cell membrane, together with the enhanced cell membrane permeability; and, eventually, cell completely disintegration.

## Introduction

*Clostridioides difficile* (previously known as *C. difficile*) (Oren and Garrity, 2016) is gram-positive anaerobic bacterium that can produce spores, accompanied by an unique off-odor (Smits et al., 2016). *C. difficile* is a conditionally pathogenic bacterium, and *C. difficile*-induced infection (CDI) accounts for 30% of antibiotic-associated diarrhea (AAD), with manifestations varying from mild diarrhea to severe complications associated with pseudomembranous colitis, toxic megacolon and death (Gao et al., 2010; Xu et al., 2017). Metronidazole and vancomycin are often used to treat CDI, but these use of these drugs leads to serious problem such as destruction of the gut microbiota, development of multidrug-resistant strains, and recurrence of infection (rCDI) (Alcalá Hernández et al., 2017; Peng et al., 2018). Recently, several new therapeutic strategies have emerged, such as fecal microbiota transplantation (FMT) for reconstruction of the gut microbiota (Juul et al., 2018), the use of octahedron iron oxide nanocrystals (Fe_3−δ_O_4_) to prevent *C. difficile* spore germination (Lee et al., 2017), the use of Manuka honey to suppress *C. difficile* biofilm formation (Piotrowski et al., 2017), and the construction of genetically engineered bacteria to target virulence genes (Saeidi et al., 2011; Bender et al., 2015); nevertheless, the use of these new therapeutic methods by the public is not widespread due to a lack of safety assessment or high cost of production. Therefore, new alternative treatments that can be easily recognized and adopted for clinical therapy are urgently required.

A large amount of clinical data has shown that some probiotics can affect CDI therapy (Mantegazza et al., 2017; Shen et al., 2017). Much attention has thus been paid to probiotics due to their great potential, particularly in medicinal applications. In fact, it has been well-known for a long time that some kinds of probiotics, such as lactic acid bacteria (LAB) and bifidobacteria, play essential roles in food fermentation, where these bacteria contribute to not only the development of the desired sensory properties in the final product but also inhibition of harmful microbial contamination (Smaoui et al., 2010). The use of probiotics to treat some diseases is becoming very popular, and CDI is one of these such disease that is being targeted. However, the antibacterial activity of specific probiotics against *C. difficile* has remained largely unknown to date, and thus, many physicians are skeptical of this probiotic therapy.

Previous studies have indicated that some probiotics, such as *Lactobacillus helveticus, Lactobacillus fermentum, Streptococcus thermophilus, Bifidobacterium longum*, and *Pediococcus pentosaceus* inhibited *C. difficile in vitro* and *in vivo*. The antibacterial effect of these strains against *C. difficile* was mainly reflected in the inhibition of growth, suppression of sporulation, and degradation of toxin (Golic et al., 2017; Wei et al., 2018; Xu et al., 2018). However, few of investigation have focused on changes at the cellular level of *C. difficile*. In this study, *C. difficile* ATCC 9689 (CD) was treated with the cell free culture supernatant of *Bifidobacterium brev*e (YH68-CFCS). A comprehensive investigation was carried on to explore the growth, spore formation, toxin production, and virulence gene expression level, especially the changes in the proton motive force (PMF), permeability, and integrity of the cytoplasmic membrane of CD, in order to illustrate the antibacterial activity of YH68-CFCS against CD *in vitro*.

## Materials and Methods

### Strains and Growth Conditions

*C. difficile* ATCC 9689 (CD) was purchased from the American Type Culture Collection, and YH68 (CGMCC No.14096) was isolated from the feces of healthy individuals (Wang et al., 2017) and provided by Jiaxing Innocul—Probiotics CO., Ltd (Jiaxing, Zhejiang, China). The strains were individually cultured in brain heart infusion (BHI) medium and de Man Rogosa Sharpe broth supplemented with 0.05% (w/v) L-cysteine (MRSC) broth for 24–48 h at 37°C anaerobically (AnaeroGenTM, Oxoid Ltd., Basingstoke, UK).

### Inhibition Zone

The cell pellets and cell-free culture supernatant (CFCS) of YH68 were collected by centrifugation at 12,000 r/min for 10 min, and the cell pellets were then resuspended in sterile saline. The antibacterial activities of the cell pellets and CFCS of YH68 against CD were tested by the Oxford cup method as reported previously (Yang et al., 2018). Briefly, 10 mL of molten solid BHI medium was poured into plates and allowed to solidify as the bottom layer, and sterilized Oxford cups were then placed on the surface of the medium. Subsequently, another 15 mL of solid BHI medium, molten and cooled to 55°C, was mixed with 600 μL of a CD culture (1 × 10^6^ CFU/mL) and poured on top of the bottom layer. The Oxford cups were removed carefully after the medium had solidified, and then, 200 μL of the cell pellets and CFCS of YH68 (3 × 10^9^ CFU/mL) was severally loaded into the holes. These plates were cultured at 37°C for 24 h after the samples had diffused for 3 h at 4°C. Sterile saline was used as the control. Finally, the diameters of the inhibition zones in all the plates were measured.

### Minimum Inhibitory Concentration (MIC) of YH68

The minimum inhibitory concentration (MIC) of YH68-CFCS toward CD was determined by a previously reported method with some modifications (Piotrowski et al., 2017). After centrifugation (12,000 r/min for 10 min), YH68-CFCS was detached and collected from bacterial culture (100 mL, 3 × 10^9^ CFU/mL). Then, 1:2, 1:4, 1:8, 1:16, 1:32, and 1:64 dilutions of YH68-CFCS were prepared with fresh BHI broth. Tubes containing 8 mL of different diluted CFCSs were inoculated with 200 μL of CD bacterial culture (1 × 10^6^ CFU/mL), incubated at 37°C for 48 h under anaerobic conditions. The cultures turbidity (OD_630nm_) was regarded as a growth indicator of CD and the lowest dilution ratio of the CFCS that exhibited no turbidity (OD_630nm_ is equal to blank control) was designated as the 1 × MIC.

### Growth and Sporulation

Two hundred microliters of fresh CD bacterial culture (1 × 10^6^ CFU/mL) was added into fresh BHI broth containing 1 × MIC of YH68-CFCS, and 1 mL culture solutions were removed from the culture broths during specific time intervals (0, 6, 12, 24, and 48 h). Half part of each sample was used to evaluate the growth with viable CFU/mL. Another 500 μL of the sample was used to determine the number of spores produced by CD, and this part was mixed with 0.5 mL ethanol (100%) for 1 h with rotation at 4°C in order to destroy the vegetative cells (Lawley et al., 2009). After that, the cell pellets were collected by centrifugation (12,000 r/min for 5 min), and washed twice in PBS (pH 7.4). The pellets were resuspended in 0.5 mL of PBS in the last wash. Spores were enumerated by serially diluting the samples in PBS and plating onto BHI agar plates with 0.1% sodium taurocholate (Sigma) (Carlucci et al., 2019). All these plates were incubated at 37°C anaerobically for 72 h, and the resultant CFU/mL values were considered to represent the relative numbers of viable spores produced.

### Toxin Production and Gene Expression

Two hundred microliter fresh bacterial culture of CD was inoculated into BHI broth (with or without YH68-CFCS) and cultivated anaerobically at 37°C for 3 h. Then, 1 mL samples were removed from the culture broths during specific time intervals (0, 6, 12, 24, and 48 h). After centrifugation (12,000 r/min), the cell pellets were collected to extract total RNA for PCR analysis of gene expression. The remaining CFCSs were filtered through a syringe filter (0.22 μm) to evaluate the content of toxin TcdA/B by an ELISA kit.

The toxin A/B protein content in the CFCSs were determined using the *C. difficile* Toxin A/B II ELISA Kit (Runyu Ltd, Shanghai, China) according to the manufacturer's instructions. Samples were diluted when necessary to obtain readings within the linear range of the standard (3–3,000 ng/mL). All the samples were tested in triplicate.

RNA was isolated from the collected CD cell pellets using the RNA-prep Pure Kit for Bacteria (TianGen Biotech, Beijing, China) according to the manufacturer's instructions. Contaminant genomic DNA was removed by two rounds of DNase treatment (DNA-free kit; Tiangen), and the final RNA yield and quality were assessed by ultraviolet (UV) absorbance and agarose gel electrophoresis, respectively. Changes in *tcdA* and *tcdB* gene expression were assessed by real-time qPCR using a method reported by Aldape et al., with some modifications, as described below with specific primers (Aldape et al., 2015).

cDNA was synthesized from each sample (500 ng) using All-in-One First-Strand cDNA Synthesis SuperMix (TransGen Biotech, Beijing, China) and amplified using TransStart Top Green qPCR SuperMix (TransGen Biotech) in a Mastercycler ep realplex system (Eppendorf, Hamburg, Germany) with 8-tube strip (Eaivelly, Shanghai, China) as follows: 30 s at 94°C, followed by 45 cycles at 94°C for 5 s, 55°C for 15 s, and 72°C for 10 s. *C. difficile* 16S rRNA served as the internal control. Relative gene expression was determined using the 2^−ΔΔ*Ct*^ method. The sample mean Ct of 16S rRNA (internal control gene) was subtracted from the sample mean Ct of the *tcdA* and *tcdB* genes (ΔCt). The ΔCt of the untreated control at 6 h was subtracted from the mean ΔCt of each experimental sample (ΔΔCt). This 2^−ΔΔ*Ct*^ method yielded the fold change in expression of the gene of interest normalized to the expression of the 16S rRNA internal control and relative to that of the untreated control at 6 h.

### Analysis of CD by Fourier-Transform Infrared (FT-IR) Spectroscopy

The cell pellets of CD (treated with 1 × MIC of YH68-CFCS or sterile saline) were collected by centrifugation at 12,000 r/min for 20 min. The pellets were then dehydrated by freeze drying and used for FT-IR spectroscopy. The IR spectra of CD were obtained by IR spectroscopy and recorded at 4°C in the wavenumber range of 4,000–400 cm^−1^ (Yang et al., 2017) with an FT-IR spectrophotometer (IR/Nicolet 6700, USA).

### Preparation of CD Cell Suspensions for YH68-CFCS Treatments

CD was inoculated into fresh BHI broth and cultivated to post-log phase at 37°C. Cell pellets were then harvested by centrifugation (12,000 r/min, 10 min) and resuspended to ~1 × 10^6^ CFU/mL in 5 mM HEPES buffer (pH 7.0) supplemented with 10 mM glucose. YH68-CFCS was added to the cell suspensions to obtain final concentrations of 1 × MIC, and then, the cells were incubated at 37°C for 3 h. The cells were monitored for changes in morphology as well as cell membrane permeability, integrity, and PMF. Cells suspensions without YH68-CFCS were used as controls.

### Analysis of the Surface Morphology of CD by Scanning Electron Microscopy (SEM)

The morphological changes in CD treated with YH68-CFCS were observed by SEM. Control and YH68-CFCS-treated cell suspensions were rinsed with 5 mM HEPES buffer (pH 7.0) and harvested by centrifugation (12,000 r/min, 10 min), followed by mixing with 2.5% (w/v) glutaraldehyde for 3 h. The precipitates were rinsed twice with 5 mM HEPES buffer (pH 7.0) for 20 min. The samples were then dehydrated by a concentration gradient (from 50 to 100%) of alcohol solutions. After dry samples were subjected to ion-sputtering coating, observation and photomicrography were then carried out with a field-emission SEM instrument (Sirion 200, USA).

### Analysis of Cell Membrane Permeability

The intra- and extracellular K^+^ and inorganic phosphate levels in CD cells were measured in order to determine whether the 1 × MIC of YH68-CFCS has an impact on cell membrane permeability (Liu et al., 2016). Control and YH68-CFCS-treated cell suspensions (2 mL) were taken at various time intervals and chilled on ice immediately. Cells were removed by centrifugation (12,000 r/min, 10 min), and the supernatants were collected for determination of extracellular K^+^ and inorganic phosphate levels. The cell pellets were resuspended in 4 mL of 5% (w/v) trichloroacetic acid and frozen overnight at −20°C. Samples were then thawed and incubated at 95°C for 10 min. Demineralized water (4 mL) was added to each sample. Cells were then removed by centrifugation (12,000 r/min, 10 min), and the intracellular K^+^ level in the supernatant was determined by inductively coupled plasma optical emission spectrometry (iCAP 7600, USA). The level of inorganic phosphate was determined by a UV Spectrophotometer (Lambda 950, China) by the Mo-Sb colorimetric method (Ames, 1966).

### Analysis of Cell Membrane Integrity

#### ATP Levels

During specific time intervals, the intra- and extracellular ATP levels were determined by a previously described bioluminescence-based method (Suzuki et al., 2005) using a microplate reader (PE & EnSpire 2300) and a CellTiter-Glo 2.0 Kit (Promega, USA).

#### UV-absorbing Materials

This experiment was performed according to a method reported by Liu et al. (2016), with some modifications. Two milliliters of cell suspension was removed from the control and YH68-CFCS-treated group every 1 h. Cell pellets were harvested by centrifugation (12,000 r/min, 10 min), and the supernatants were filtered through a syringe filter (0.22 μm). The absorbance of the supernatants was recorded at OD_260_ and OD_280_ using a UV spectrophotometer (Lamda 950, China).

#### Confocal Laser Scanning Microscopy (CLSM)

Confocal laser scanning microscopy (CLSM) analyses using the fluorescent indicators SYTO 9 and propidium iodide (PI) were performed as described by Liao et al. (2010) to assess the damage caused by YH68-CFCS to the CD cell membranes within a short time. Two hundred microliters of cell suspensions from the control and YH68-CFCS-treated groups were mixed with 200 μL of a dye mixture containing SYTO 9 and PI (final concentrations of 6 and 30 μM, respectively) from a LIVE/DEAD® BacLightTM Bacterial Viability Kit (Promega, USA) for 15 min at room temperature in the dark. Then, 10 μL of the final mixed cell suspension was trapped between a slide and an 18 mm square coverslip and examined under a super-resolution multiphoton confocal microscope (TCS SP8 STED 3X, Leica, Germany) with a 500 × objective oil immersion lens.

### Analysis of the Proton Motive Force (PMF) of the Cytoplasmic Membrane

#### Transmembrane Electrical Potential (Δψ)

The Δψ of the cytoplasmic membrane was monitored with the fluorescent probe 3,3-dipropylthia-dicarbocyanine iodide [DISC3(5)] (Sigma-Aldrich, USA) (Molenaar et al., 1991; Castellano et al., 2003). CD cell suspensions were mixed with 0.5 μM DISC3(5), and then, nigericin (1 μM) (Aladdin, USA), valinomycin (1 μM) (Sigma-Aldrich, USA), or YH68-CFCS (final concentration, 1 × MIC) was added into the cell suspensions. An untreated cell suspension was used as the control. A microplate reader (PE EnSpire 2300) was used to measure the fluorescence value at excitation and emission wavelengths of 500 and 690 nm, respectively.

#### Transmembrane pH Gradient (Δ pH)

2',7'-bis-(2-carboxyethyl) 5(and-6)-carboxyfluorescein (BCECF) (Sigma-Aldrich, USA) was used as a fluorescent pH indicator and added into the cells solution containing 10 mM glucose, to detect the change of ΔpH (alkaline on the inside) (Molenaar et al., 1991; Castellano et al., 2003). Fluorescence values at excitation and emission wavelengths of 502 and 525 nm were, respectively, determined.

### Statistical Analysis

Each assay was repeated three times independently and the data were expressed as the means ± standard deviations. The differences between the groups were examined by the Student's *t*-test or the one-way analysis of variance [ANOVA] followed by Tukey's Multiple Comparison test using Minitab 16.2.3 software (Minitab Inc., State College, PA). A *P* < 0.05 was considered statistically significant.

## Results

### MIC of YH68-CFCS

Inhibition zone assay was carried out to evaluate the main antibacterial components of YH68. The inhibition zone of the CFCS reached 19.10 ± 0.54 mm, which was much larger than those of the cell pellets (7.25 ± 0.21 mm) and sterile saline (7.00 ± 0.00 mm) (*p* < 0.05). This finding indicated that the YH68-CFCS contained the main antibacterial substances. Thus, the YH68-CFCS was used for follow-up experiments. Then, the double-dilution assay was used to determine the 1 × MIC of YH68-CFCS. It showed that the double-dilution ratio of YH68-CFCS (3 × 10^9^ CFU/mL) was the 1 × MIC and this diluted concentration exhibited no turbidity (no CD growth). This diluted concentration of YH68-CFCS was regarded as the 1 × MIC and used to treat CD.

### Effects of YH68-CFCS on the Growth and Spore Production of CD

**Figure 1:**
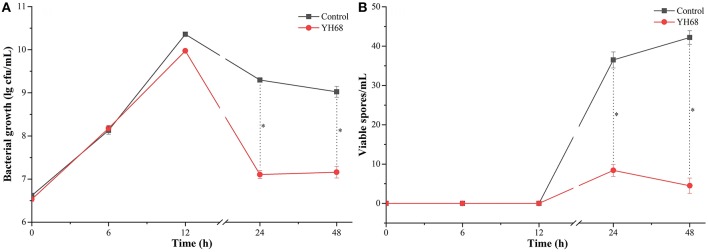
Bacterial growth and viable spores of *C. difficile* ATCC 9689 at different time points. **(A)** Bacterial growth lg cfu/mL; **(B)** Viable spores/mL (control and YH68 at the same time point; ^*^P < 0.05; Student's *t*-test).

Based on the measurement of physiological indexes, the inhibitory effect of YH68-CFCS on CD was explored. The growth of CD in the presence of YH68-CFCS and in the control exhibited a steady increase within 12 h (Figure 1). Then, this increasing trend was terminated and replaced by a decreasing trend until 48 h. Spore production by CD were detected at 24 and 48 h. The cell density and spore production of CD treated with YH68-CFCS reached 7.11 ± 0.09 lg CFU/mL and 8.40 ± 1.53 spores/mL, respectively, at 24 h and 7.16 ± 0.14 lg CFU/mL and 4.50 ± 1.95 spores/mL, respectively, at 48 h. Both the values for the CD treated with YH68-CFCS were less than those for the control (the cell density and spore production reached 9.30 ± 0.03 lg CFU/mL and 36.50 ± 2.04 spores/mL, respectively, at 24 h and 9.02 ± 0.12 lg CFU/mL and 42.20 ± 1.73 spores/mL, respectively, at 48 h) (*p* < 0.05), indicating that the CFCS of YH68 at 1 × MIC has a stronger inhibitory effect on the growth and spore production of CD.

### Effects of YH68-CFCS on the Toxin Production and Gene Expression in CD

**Figure 2:**
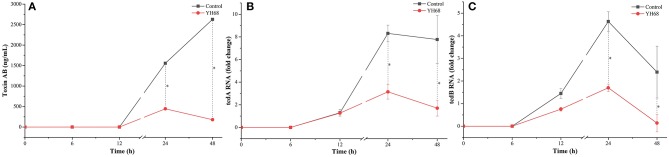
Toxin production and tcdA/B gene expression of *C. difficile* ATCC 9689 at different time points. **(A)** Toxin production; **(B)** tcdA gene expression; **(C)** tcdB gene expression (control and YH68 at the same time point; ^*^P < 0.05; Student's *t*-test).

CD were treated with YH68-CFCS to explore whether their toxin production was inhibited and their virulence gene expression levels were downregulated. The result showed that toxin A/B was not detected before 12 h, whereas the expression of *tcdA* and *tcdB* was detected at an earlier timepoint (Figure 2). Data showed that toxin A/B production in the YH68-CFCS treatment group reached 445.62 ± 7.24 ng/mL at 24 h and 179.45 ± 0.00 ng/mL at 48 h, which were much lower than the values for the control (1556.38 ± 5.35 ng/mL at 24 h, 2628.74 ± 3.62 ng/mL at 48 h) (*p* < 0.05). The gene expression levels of *tcdA/B* in the CD treated with YH68-CFCS decreased, and the fold change for *tcdA* was 3.14-fold (control was 8.31-fold) at 24 h and 1.70-fold (control was 7.77-fold) at 48 h (*p* < 0.05); for *tcdB*, the fold change was 1.70-fold (control was 4.63-fold) at 24 h and 0.14-fold at 48 h (control was 2.40-fold) (*p* < 0.05). Based on the toxin production and gene expression levels of *tcdA/B* in CD, the CFCS of YH68 at 1 × MIC notably reduced toxin production and decreased virulence gene expressions, which indicated that YH68-CFCS had an inhibitory effect on CD in terms of toxin production.

### FT-IR Analysis of CD Treated With YH68-CFCS

**Figure 3:**
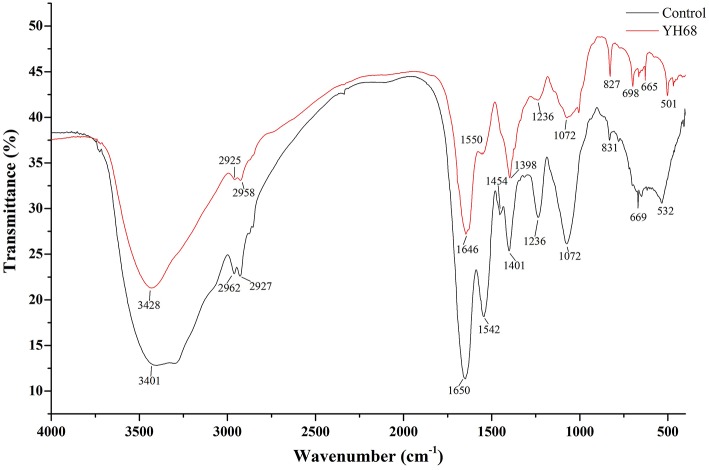
FT-IR spectra of *C. difficile* ATCC 9689.

FT-IR spectral analysis was used to explore the main chemical compound changes of CD cells with or without YH68-CFCS treatment. The FT-IR spectra of cell pellets from the control and YH68-CFCS treatment groups are shown in Figure 3. Both of spectra have broad, intense bands at 3,401–3,428 cm^−1^, corresponding to the stretching vibrations of the O-H and N-H bonds. The strong bands at 2,925–2,962 and 1,646–1,650 cm^−1^ indicates the presence of CH (-CH_2_, -CH_3_) and C = C stretching vibrations, respectively. The strong bands at 1,454–1,550 cm^−1^ and 1,398–1,401 cm^−1^ indicates the presence of a benzene skeleton and hetero aramid vibrations, respectively. The absorption peaks at ~1,072–1,236 and 665–831 cm^−1^ are indicative of bending vibrations (P = O, P = S) and a C-H bending vibration in the benzene ring, respectively. Furthermore, the FT-IR spectra had a number of absorption peaks at 501–532 cm^−1^ (indicative of a C-Br compound group). Previous studies have demonstrated that the strong bands at 2,800–3,000 cm^−1^ correspond to the absorption peaks of fatty acids in cell membranes; those at 1,500–1,800 cm^−1^ represents the characteristic peaks of amino acid and amino acid; those at 1,200–1,500 cm^−1^ demonstrates the presence of compounds containing protein and phosphoric acid groups; and those at 900–1,200 cm^−1^ are the absorption peaks of polysaccharides in the cell wall (Moen et al., 2005; Zoumpopoulou et al., 2010).

In terms of peak shifts, the absorption peaks at 1,454 and 1,542 cm^−1^, which are present in the spectrum of the control, are absent in the spectrum of CD treated with YH68-CFCS. In terms of peak areas, three marked changes were observed for the peaks at 1,500–1,800, 1,200–1,500, and 900–1,200 cm^−1^, which indicated that YH68-CFCS induced significant changes in the structures of CD cells. These data demonstrated that YH68-CFCS caused serious damage to the CD cells, including changes in the cell membranes and walls.

### Effect of YH68-CFCS on the Cell Structures of CD

**Figure 4:**
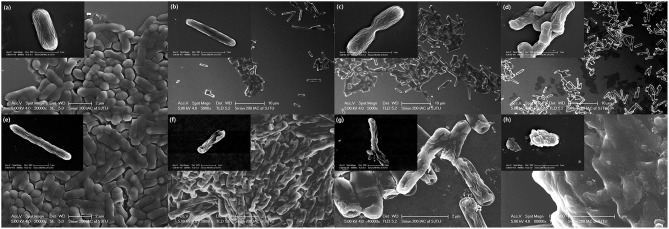
Morphology of *C. difficile* ATCC 9689 observed by scanning electron microscopy. **(a–d)**: Control at 0, 1, 2, and 3 h; **(e–h)**: treated with YH68-CFCS for 0, 1, 2, and 3 h.

Changes in cell morphology are the most intuitive manifestation. SEM was used to determine whether YH68-CFCS greatly changed the cell structures of CD. The cells of CD treated with YH68-CFCS exhibited a shrunken shape, cytoplasm leakage and dissolved cell walls, in contrast to the morphology of intact cells of the control, over time (Figure 4). Specifically, the cell morphology of CD changed greatly, including changes in cell surface morphology and dissolution of the cell wall at 2 and 3 h. These SEM images are visual proof that YH68-CFCS induced CD cell damage within 3 h.

### Effect of YH68-CFCS on Cell Membrane Permeability

**Figure 5:**
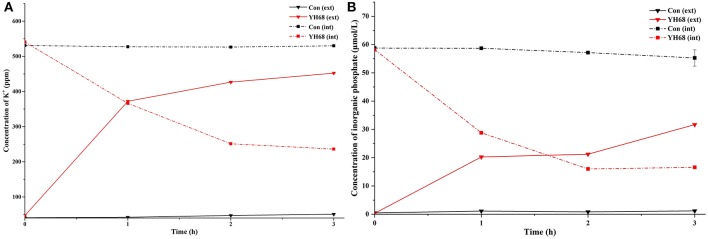
Intracellular (int) and extracellular (ext) K+ **(A)** and inorganic phosphate **(B)** levels in *C. difficile* ATCC 9689 cells.

The K^+^ levels and inorganic phosphate contents in CD cells varies greatly after their cell membrane permeability was changed and destroyed by YH68-CFCS. The intra and extracellular K^+^ levels and inorganic phosphate contents of CD were measured in order to determine whether YH68-CFCS had an impact on cell membrane permeability (Figure 5).

The K^+^ levels of the CD cells in the control were stable (intracellular K^+^ was 530 ppm, extracellular K^+^ was 45 ppm) over the 3 h period, whereas the intracellular K^+^ levels of the CD cells treated with YH68-CFCS decreased significantly (236.2 ppm at 3 h) (*p* < 0.05); subsequently, the extracellular K^+^ levels increased and reached 452.4 ppm at 3 h. A similar change was observed in the inorganic phosphate contents of CD. The intracellular inorganic phosphate content of the CD cells in the control stabilized at 0 μmol/L (the extracellular content stabilized at 58 μmol/L) over time, whereas the intracellular content of the CD cells treated with YH68-CFCS decreased to 16.59 μmol/L (the extracellular content increased to 31.68 μmol/L) at 3 h (*p* < 0.05). These results revealed that YH68-CFCS induced a significant change in the cell membrane permeability of CD, subsequently resulting in leakage of intracellular K^+^ and inorganic phosphate and eventually leading to CD damage.

### Effect of YH68-CFCS on Cell Membrane Integrity

**Figure 6:**
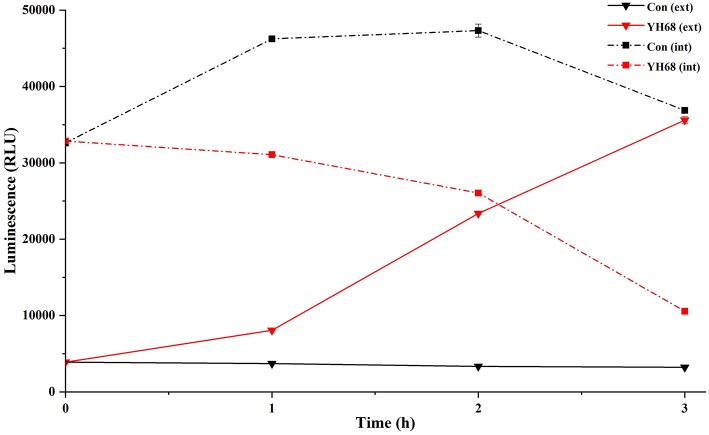
Intracellular (int) and extracellular (ext) ATP levels in *C. difficile* ATCC 9689 cells.

**Figure 7:**
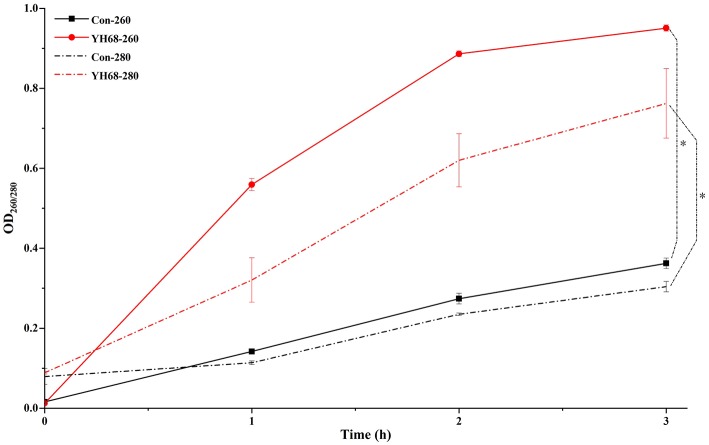
Extracellular UV-absorbing materials from *C. difficile* ATCC 9689 cells (^*^P < 0.05; one-way analysis of variance [ANOVA] followed by Tukey's Multiple Comparison test).

**Figure 8:**
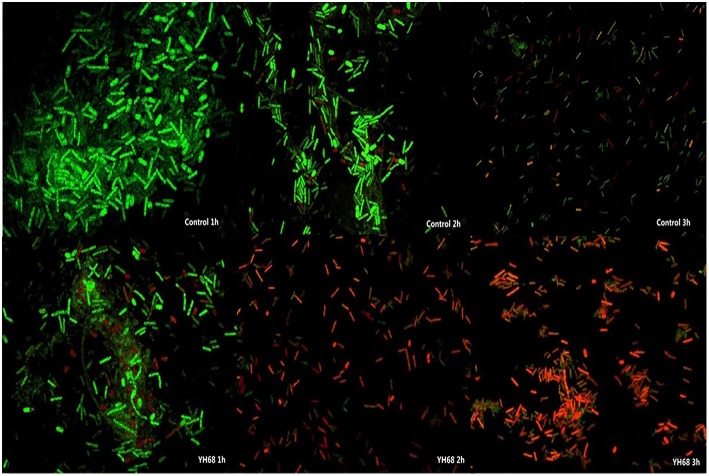
Confocal laser scanning micrographs of *C. difficile* ATCC 9689 treated with YH68-CFCS for 1, 2, 3 h.

Cell membrane integrity of CD was destroyed by YH68-CFCS, followed by the formation of several pores in the cell membrane, accompanied by a release of ATP, nucleic acid and protein substances from the interior of cells. The changing levels of ATP (luminescence value) and UV-absorbing materials (OD_260/280_ value) from CD cells were determined and regarded as indicators of cytolysis and random pore formation (Figures 6, 7). For the control, the intra- and extracellular ATP levels changed slightly, and the intra- and extracellular luminescence values stabilized at 40,000 and 3,500 RLU, respectively, over 3 h, indicating that the cell membrane of CD was almost intact. However, the intracellular luminescence value of the CD cells treated with YH68-CFCS decreased dramatically and reached 10,566 RLU at 3 h (*p* < 0.05), and simultaneously, the extracellular luminescence level increased to 35,604 RLU (*p* < 0.05), indicating that the CD cell membrane was damaged, which was accompanied with leakage of intracellular ATP. Meanwhile, in contrast to the control, nucleic acids and proteins were discharged from the CD cells treated with YH68-CFCS, exhibiting an increase in absorbance value over 3 h (Figure 7). These results suggested that the cell membrane of CD was damaged by YH68-CFCS in a short time period. Subsequently, CLSM coupled with the fluorescent dye SYTO 9 and PI was used to further confirm the damage of CD cells from an intuitively survival rates perspective. In general, all the bacterial cells can be stained by SYTO 9. Only cells that their membranes are broken can be penetrated by PI, which can reduce the intensity of SYTO 9 fluorescence (Moussa et al., 2007). Normal bacterial cell membranes are integrity and can exclude PI but allowed the entry of SYTO 9, accompanied with the excitation of green fluorescence; however, bacterial cells have damaged membranes can be stained by PI with the excitation of red fluorescence. There was a gradual change between green and red fluorescence intensity of CD cells after treated with YH68-CFCS over time (Figure 8). The control cells (treated with 85% sterile saline) excited transparent green fluorescence with little red fluorescence over the 3 h period, indicating the infiltration of SYTO 9 and the impediment of PI. In contrast, the intensity of the green fluorescence in CD cells treated with YH68-CFCS decreased after 2 h, while the intensity of the red fluorescence increased, accounted for a large proportion (compared with that at 1 h) of treated cells suffered a loss of membrane integrity. Three hours later, almost all of the treated CD cells exhibited red fluorescence, not green. These data above revealed that the CD cell membrane damaged under the treatment of YH68-CFCS, and this destruction was a progressive process.

### PMF Dissipation Induced by YH68-CFCS

**Figure 9:**
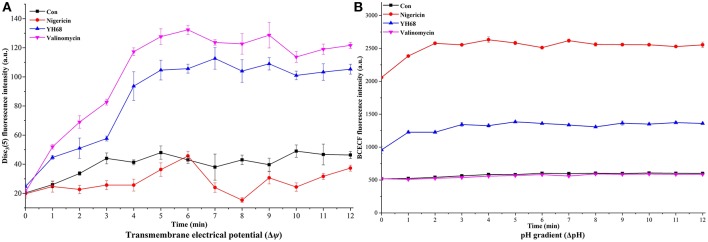
Transmembrane electrical potential (Δψ) **(A)** and pH gradient (ΔpH) **(B)** in *C. difficile* ATCC 9689.

Destruction of cytoplasmic membranes of CD cells, reflected in the PMF dissipation, including the changes of Δψ and ΔpH. The effects of YH68-CFCS on Δψ and ΔpH were evaluated in order to explore whether YH68-CFCS has an influence on the PMF of the cytoplasmic membranes of CD cells (Figure 9). Valinomycin (1.0 μM) was added and used as a mobile ion carrier, causing a rapid and complete loss of Δψ. Nigericin (1.0 μM) was used as a channel forming agent, which contributed to the maintenance of Δψ in CD cells. During the treatment of YH68-CFCS, the fluorescence value of the CD cells was constantly changing and reached a peak of 112. 67 (a.u.) at 7 min. These data demonstrated that YH68-CFCS possessed the similar function (like valinomycin), inducing a rapid and complete depolarization of the cytoplasmic membrane. Whereas, this phenomenon was disappeared after the addition of valinomycin. This result indicated that YH68-CFCS could separate induce the dissipation of the Δψ.

In the presence of intracellular BCECF fluorescence, the change of the ΔpH was determined. The changes of fluorescence intensity corresponds to the loss of BCECF in the CD cells. After the addition of nigericin (1.0 μM), a rapid collapse of the ΔpH was caused. The pH values of internal and external environment experienced a change and achieved a final balance. Valinomycin (1.0 μM) was added in order to ensure that ΔpH was the sole component of the PMF. The fluorescence intensity shown a slight decline after the addition of valinomycin (Figure 9B), suggesting there was an interaction or interconversion between the membrane potential and the pH gradient. However, the intensity of fluorescence was enhanced rapidly over 3 min after nigericina was added. A similar increasing trend was also observed after the addition of YH68-CFCS. The ascending pH value of internal environment was primarily due to the efflux of H^+^. These data suggested that the collapse of ΔpH was attributed in part to YH68-CFCS. Meanwhile, this result proved that YH68-CFCS could induce the dissipation of both components in the PMF.

## Discussion

There are several precedents for using probiotics to prevent or treat CDI in clinical therapy (Goldstein et al., 2017; Mills et al., 2018). A recent clinical investigation revealed that probiotics and antibiotics used in combination greatly reduced the incidence of CDI (Shen et al., 2017). Probiotics have long been used by the public due to their generally recognized as safe (GRAS) status as well as their inherent natural properties, such as antibacterial activity (Leite et al., 2015). The antibacterial activity of probiotics against pathogenic bacteria has many different modes, including cell adhesion, nutrient competition, ecological niche competition and the secretion of antibacterial substances (Barbosa et al., 2015; Gonzalez-Ochoa et al., 2017). *Bifidobacterium* species, as representative probiotics, are widely used in the fields of food fermentation and biomedicine (Jena et al., 2017). In this study, the antibacterial activity of YH68 mainly originated from the CFCS.

An investigation from Liu et al. (2018) revealed that the molecular characterization of *C. difficile* isolates in China from 2010 to 2015 is diversity and ribotype 001 (9689) is one of the most prevalent (Liu et al., 2018). Therefore, *C. difficile* ATCC 9689 was used as a reference strain in this study. In terms of inhibition, the concentration of YH68-CFCS at 1 × MIC had a marked inhibitory effect on the growth of CD as well as on spore production, which is the immediate cause of the recurrence CDI. These effects are not limited to YH68-CFCS; Ratsep et al. (2017) found that *L. plantarum* DSM 21379 combined with xylitol strongly inhibited the spore germination of CD and subsequently reduced the infection rates. Previous research has indicated that expression of the *spo0A* gene, which can control spore production, was downregulated upon treatment with antibiotics (Aldape et al., 2015). In our study, the inhibitory effect of YH68-CFCS on spore production of CD was similar to that of antibiotics. In addition, the production of toxin A/B by CD was also inhibited by YH68-CFCS, which was most likely associated with some active substances in the CFCS. Wei et al. (2018) found that the toxin A/B content of CD was reduced by the CFCS of *B. longum* JDM301, especially the level of toxin A, which was significantly reduced by organic acids in the CFCS. However, the production of toxin A/B is directly controlled by the virulence genes *tcdA* and *tcdB* of CD. Yun et al. (2014) found that a decrease in the transcriptional level of *tcdA* and *tcdB* genes in *C. difficile* RT027 induced by 10 μg/mL of cell extract of *Lactobacillus acidophilus* GP1B, followed by a rapidly decreasing in toxin production. Kolling et al. (2012) found that the CFCS of *S. thermophilus* led to the downregulation of *tcdA* expression in *C. difficile* TL24, mainly due to the lactic acid in CFCS (concentration was 10 mM). Previous studies indicated that the expression levels of *tcdA/B* associated with the production (*luxS*) of autoinducer-2 (AI-2) in *C. difficile*, which is an important component of a quorum sensing (QS) system in *C. difficile* (Lee and Song, 2005; Yun et al., 2014). CFCS or extracts of probiotics decreased the pathogenicity of *C. difficile* by inhibiting QS, subsequently downregulating the expression level of QS-regulated toxin genes (*tcdA* and *tcdB*) (Yun et al., 2014). Data from the real-time qPCR assay in this study showed that the expression of *tcdA/B* began before toxin A/B production by CD. The fold change in *tcdA* and *tcdB* expression in CD treated with YH68-CFCS was 1.70- and 0.14-fold, respectively, at 48 h, which was lower than that of the control (*tcdA* was 7.77-fold and *tcdB* was 2.40-fold at 48 h). These results demonstrated that the expression level of *tcdA/B* in CD was significantly (*p* < 0.05) reduced by YH68-CFCS, thereby inhibiting the production of toxin A/B. These dominant results revealed the inhibitory effect of YH68-CFCS against CD, including effects on growth, spore production, toxin production and virulence gene expression.

In terms of destruction, the changes in CD cells triggered by YH68-CFCS at the cellular level were explored in this study. It is well-known that the antibacterial activity of probiotics against pathogenic bacteria is mainly due to the antibacterial substances secreted by probiotics, which directly induce the destruction of pathogenic bacterial cell structures (Liu et al., 2016). Significant changes were observed in the proteins and amino acids of the cell membranes as well as in the polysaccharide skeletons of the cell walls. Furthermore, visual proof was obtained by SEM, which demonstrated substantial changes in the cell morphology of CD cells treated with YH68-CFCS, including changes in cell surface morphology, wrinkle formation in cells, and eventual dissolution of cell membranes within 3 h. Liu et al. (2016) found that bifidocin A produced by *B. animalis* 04 induced severe deformation of *E. coli* 1.90 cells, especially of the cell membranes, in a short time period. Thus, the changes in and damage to the cell membranes played a critical role in the destruction of CD cells in the present study. Leakage of a large amount of intracellular K^+^, inorganic phosphate, ATP, nucleic acid and protein from CD cells treated with YH68-CFCS was observed, indicating severe destruction of cell membrane permeability and integrity. The fluorescent probe DISC_3_ (5) and fluorescent pH indicator BCECF were used to determine the effect of YH68-CFCS on the PMF of the cytoplasmic membrane, including the effects on the indexes of Δψ and ΔpH. In addition, these results revealed that YH68-CFCS was responsible for the partial collapse of ΔpH and indicated that YH68-CFCS was able to dissipate both components of the PMF. These effects are clearly associated with the efflux of K^+^, inorganic phosphate, ATP, and UV-absorbing materials upon treatment with YH68-CFCS.

Previous studies have demonstrated that the antibacterial effect of probiotics is mainly associated with metabolites such as organic acids (mainly lactic-, acetic-, propionic-, sorbic-, and benzoic-acids), hydrogen peroxide, diacetyl, ethanol, phenols, proteinaceous compounds and exopolysaccharides as well as bacteriocins that are produced during bacterial growth (Bajpai et al., 2016). These antibacterial substances can directly affect pathogenic bacteria, mainly by causing cell damage. However, another mode of antibacterial activity was reported in a previous study; Saraoui et al. (2016) found that the antibacterial activity of *Lactococcus piscium* CNCM I-4031 against *Listeria monocytogenes* occurred mainly via direct interaction between the two species. In this study, it was found that the CFCS of YH68 was the main contributor to the antibacterial activity against *C. difficile* ATCC 9689.

Probiotics exhibit a desirable advantage over antibiotics in clinical therapy. Currently, antibiotics are preferred for the treatment of CDI, which has led to an alarming increase in the emergence of highly resistant strains and perturbance of the intestinal flora. Zarandi et al. (2017) found that CAZ or VAN combined with CAZ stimulated toxin A/B production in CD compared with the production in the control. In this study, the production of toxin A/B in CD cells treated with YH68-CFCS decreased greatly at 24 and 48 h, indicating that YH68-CFCS is better than some antibiotics in terms of inhibition of toxin production. Previous studies have focused on the change in the toxin production induced by derivatives from probiotics at the different culture conditions (Trejo et al., 2010; Bolla et al., 2013; Valdes-Varela et al., 2016; Wei et al., 2018), however, few reports about the effect of time interval on the toxin production, especially the different time points. Here, we obtained the details about the effects of YH68-CFCS on the toxin production of CD at the different time intervals. In addition, there was a difference in gene expression between YH68-CFCS and antibiotics treatments. Gerber et al. (2008) found that the expression levels of *tcdA* and *tcdB* were enhanced by MTR, VAN, and LZD at sub-inhibitory concentrations in comparison with the levels observed in the control. However, our data indicated that the gene expression level of *tcdA/B* in CD treated with YH68-CFCS was significantly downregulated (*p* < 0.05). In terms of the inhibition of toxin production and gene expression, YH68-CFCS possesses more advantages than antibiotics for the treatment of CDI; theoretically, YH68-CFCS might alleviate the damage caused to intestinal cells and reduce the recurrence rate of CDI. In this study, CD was significantly inhibited and destroyed by YH68-CFCS at 1 × MIC. The antibacterial activity of YH68-CFCS against CD involved the inhibition of growths, spore and toxin production as well as virulence genes expression; meanwhile, changes at the cellular level were reflected in the dissipation of the PMF of the cytoplasmic membrane, increased in membrane permeability, pore formation in the cell membrane, altered membrane integrity and complete cell disintegration. All these results suggested that *C. difficile* ATCC 9689 were destroyed by *Bifidobacterium breve* YH68-CFCS, mainly depended on the harmful intervention of physiological activities and a direct damage on cell morphology.

## Data Availability

The raw data supporting the conclusions of this manuscript will be made available by the authors, without undue reservation, to any qualified researcher.

## Author Contributions

JY and HY designed experiments, analyzed the data, and wrote the manuscript. JY performed the experiments.

### Conflict of Interest Statement

The authors declare that the research was conducted in the absence of any commercial or financial relationships that could be construed as a potential conflict of interest.
